# Targeted cellular ablation based on the morphology of malignant cells

**DOI:** 10.1038/srep17157

**Published:** 2015-11-24

**Authors:** Jill W. Ivey, Eduardo L. Latouche, Michael B. Sano, John H. Rossmeisl, Rafael V. Davalos, Scott S. Verbridge

**Affiliations:** 1 Department of Biomedical Engineering and Mechanics, Virginia Tech-Wake Forest University, Blacksburg, VA, 24061; 2 Department of Radiation Oncology, Division of Radiation Physics, Stanford University School of Medicine, Stanford, CA 94305; 3 Neurology and Neurosurgery, Virginia-Maryland College of Veterinary Medicine, Blacksburg, VA, 24061; 4 Comprehensive Cancer Center, Wake Forest Baptist Medical Center, Winston-Salem, NC, 27157.

## Abstract

Treatment of glioblastoma multiforme (GBM) is especially challenging due to a shortage of methods to preferentially target diffuse infiltrative cells, and therapy-resistant glioma stem cell populations. Here we report a physical treatment method based on electrical disruption of cells, whose action depends strongly on cellular morphology. Interestingly, numerical modeling suggests that while outer lipid bilayer disruption induced by long pulses (~100 μs) is enhanced for larger cells, short pulses (~1 μs) preferentially result in high fields within the cell interior, which scale in magnitude with nucleus size. Because enlarged nuclei represent a reliable indicator of malignancy, this suggested a means of preferentially targeting malignant cells. While we demonstrate killing of both normal and malignant cells using pulsed electric fields (PEFs) to treat spontaneous canine GBM, we proposed that properly tuned PEFs might provide targeted ablation based on nuclear size. Using 3D hydrogel models of normal and malignant brain tissues, which permit high-resolution interrogation during treatment testing, we confirmed that PEFs could be tuned to preferentially kill cancerous cells. Finally, we estimated the nuclear envelope electric potential disruption needed for cell death from PEFs. Our results may be useful in safely targeting the therapy-resistant cell niches that cause recurrence of GBM tumors.

Cancer therapies have historically focused on targeting the bulk of a tumor with surgical resection, or the highly proliferative phenotypic characteristics of cancer cells with chemotherapy. These are generally combined with radiation therapy to induce physical damage to tumor cells. More recently molecularly targeted therapies have gained attention^1^,^2^ which target specific mutations such as Her2 overexpression in breast cancer. However each of these treatments has significant downsides for the quality of the patient’s life and duration of survival. Chemotherapy and radiation result in relatively indiscriminant damage to normal cell types. In the case of brain cancer this leads to radiation necrosis, pseudo-progression^3^ and cognitive defects in 20–50% of patients undergoing whole brain radiotherapy^4^. Surgery fails to remove disseminated invasive cells that lie beyond the surgical resection border, while targeted therapies place a selection pressure leading to the emergence of therapy-resistant cells, both of which may lead to tumor recurrence and ultimately patient death. Especially in the case of glioblastoma multiforme (GBM), a highly aggressive and invasive form of brain cancer, the tumor is characterized by multiple levels of heterogeneity^5^,^6^,^7^, leading to predictable recurrence after initial treatment rounds.

The intratumoral heterogeneity of GBM is responsible, at least in part, for the failure of both conventional and targeted therapies to greatly extend the lifespan of patients diagnosed with GBM^1^,^2^,^8^,^9^. These tumors are made up of cells that vary greatly in their genetic, transcriptional, and phenotypic profiles, across varying microenvironmental niches^5^,^10^. This microenvironmental heterogeneity also manifests itself in physical differences in cells in the tumoral space. For example, GBM is characterized by an invasive front of cells that spread along white matter tracts, take on a different morphology, and perhaps also adopt a different mechanical phenotype to accomplish invasion^11^. The extension of tumor cells into the surrounding brain parenchyma contributes significantly to the failure of surgery as a treatment method, however there is no method to target these infiltrative cells preferentially without damaging critical surrounding structures such as astrocytes, neurons and blood vessels^12^. It remains an open challenge for GBM, as for all highly malignant tumors, to find a treatment that may preferentially target malignant cells, yet not succumb to resistance mechanisms that plague all existing therapies.

To address the need for a therapy to preferentially target malignant cells, we have developed a cellular ablation method using pulsed electric fields (PEFs). In PEF therapy, pulses are applied through electrodes inserted directly into a tumor, establishing an electric field across a well-defined tissue volume. Cells polarize in the presence of this external electric field resulting in an elevated transmembrane potential (TMP). If the TMP breaches a critical threshold, transient nanoscale pores form in the plasma membrane, which allow large molecules to traverse across the lipid bilayer^13^. This phenomenon, known as reversible electroporation^14^, is a well-established method used in aiding drug delivery, or for delivery of genetic material^15^,^16^. Beyond another critical TMP threshold, typically 1 V, irreparable damage occurs, preventing the resealing of these pores, which leads to cell death. This mechanism of cell death has been leveraged as a treatment modality known as irreversible electroporation (IRE), which has been applied to treat a variety of cancers^17^,^18^.

IRE offers the major advantages of sparing sensitive structures such as major blood vessels^18^ and the extracellular matrix (ECM). IRE treatments produce ablations with a sub-millimeter transition between unaffected and necrotic tissue^19^,^20^ and the ablation area can be readily predicted through mathematical modeling^21^. Treatments using long (~100 μs) pulses have been shown to induce death through disruption of the cell membrane^22^. However short to mid-range pulses (0.1–10 μs) remain largely unexplored for mammalian cells, and it is theorized that these pulses may provide access for electrically manipulating organelles^22^. For pulse lengths shorter than the plasma membrane charging time (~1 μs) the majority of charge buildup is no longer confined to the plasma membrane^23^. Instead, fast rise-times cause the potential drop to occur within the cell’s interior. We have developed high-frequency irreversible electroporation (HFIRE)^24^,^25^, which uses bipolar square waves of 1 μs pulses delivered in a rapid burst (Fig. 1a), to explore the possibility of organelle targeting.

Despite the genetic heterogeneity within tumors that acts as a hindrance to most therapies, PEF treatments that target physical characteristics of malignant cells may provide a more effective means of targeting the most malignant sub-populations of such tumors. Cellular morphology, particularly cell size and nuclear-to-cytoplasm ratio (NCR)^26^,^27^,^28^, as well as cellular electrical properties^29^ are known to differ between normal and tumor cells. We hypothesize that phenotypic characteristics of malignant and normal cells present in the GBM microenvironment vary sufficiently to provide targets for cell-specific ablation with properly tuned PEFs. Using 3D micro-engineered mimics of normal and malignant brain tissues, with experimentally defined ECM composition, we investigated cell-specific response to a range of pulse frequencies, to determine the extent to which either IRE or HFIRE can target specific morphological cellular characteristics within a heterogeneous microenvironment.

## Results

### Cell size selectivity of pulsed electric fields

**Figure 1:**
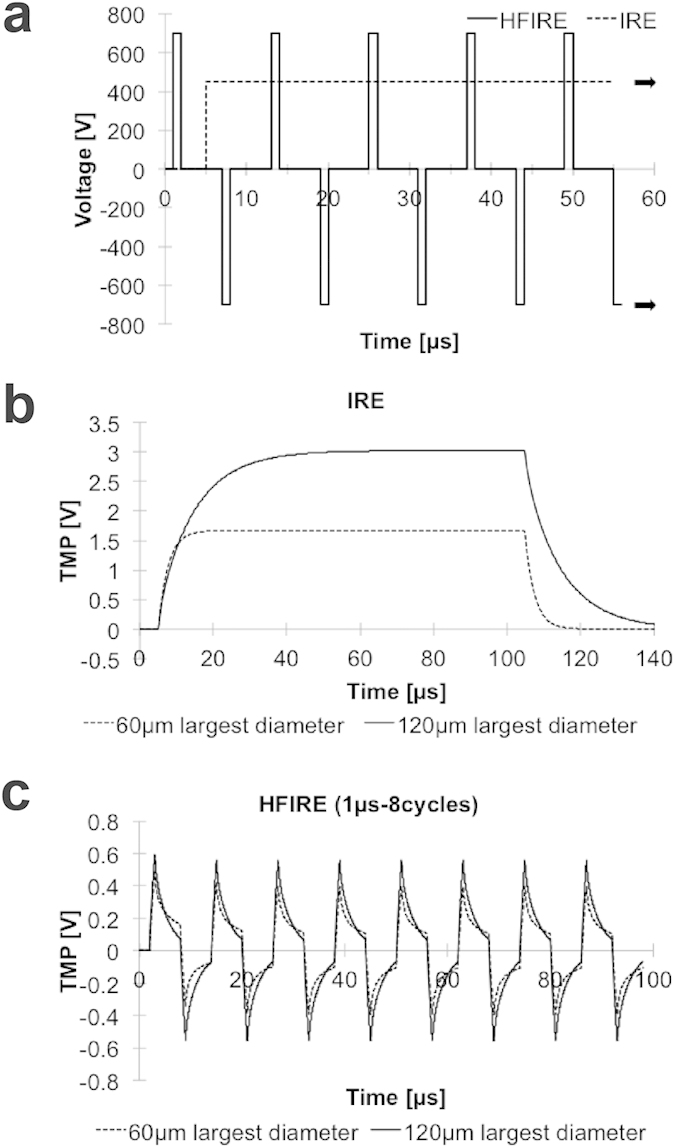
Finite element modeling using two pulse waveforms predicts IRE is cell size dependent while HFIRE is cell size independent. (**a**) Simulated unipolar 100 μs IRE waveform and bipolar 1 μs HFIRE waveform. (**b**) Calculated cellular TMP response for two different cell sizes exposed to an IRE waveform applying 500 V/cm shows TMP size dependence. (**c**) HFIRE pulse waveform response shows no TMP cell size dependence at 500 V/cm. TMP values were calculated at a point where the cell membrane is perpendicular to the direction of the electric field.

Single cell responses to electric field pulses were simulated with finite element modeling. Simulated TMP changes in response to modeled IRE pulses (Fig. 1a) are highly dependent on cell size (Fig. 1b). In contrast, cells exposed to HFIRE pulses do not show significant TMP variation with cell size in these models (Fig. 1c). It should be noted that the maximum TMP reached by both cell types when exposed to HFIRE pulses is significantly lower than the TMP for IRE pulses. This is because the 1 μs HFIRE pulse durations are shorter than the cell membrane charging time. If the pulse duration was progressively increased, the TMP would approach the values calculated in the IRE case^24^.

**Figure 2:**
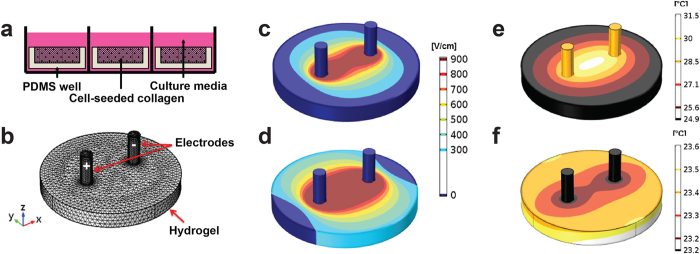
Finite element models predict the electric field and thermal distributions within hydrogel platforms. (**a**) Engineered 3D collagen hydrogels are made by adding cell-seeded collagen (0.2% or 2% w/w) into PDMS wells of controlled geometry. They are kept in a well plate under cell culture conditions with nutrients supplied by culture media. (**b**) Mesh used to calculate the electric field distribution within the tissue mimics illustrates the experimental setup for therapy testing. Electric field (V/cm) iso-contours when (**c**) 450 V and (**d**) 700 V pulses are simulated. (**e**) Temperature isocontours immediately post-therapy (50 pulses of 700 V) show a maximum temperature rise of 12 °C above room temperature. (**f**) Temperature isocontours one minute post-therapy confirm that cells are not exposed to any long-term thermal effects as a result of IRE or HFIRE pulses.

To experimentally explore the effect of cell size on electric field thresholds for cell death, we tuned the mechanical and chemical structure of the tumor microenvironment using a three-dimensional GBM hydrogel tumor model (Fig. 2a, Fig. S1) to then be used as a therapy-testing platform (Fig. 2b). We determined the lethal electric field threshold by simulating the electric field within the hydrogels during pulse exposure, at the two experimental voltages, using finite element modeling (Fig. 2c,d). These simulations reveal the change in expected lesion shape as a function of voltage, evolving from a peanut to a circular shape as the electric field magnitude increases. Finite element modeling of treatment-induced temperature distribution in the hydrogel demonstrates that cellular damage does not occur through thermal effects, as cells are not exposed to temperatures above physiological levels (Fig. 2e), with no long-term temperature increases evident (Fig. 2f).

**Figure 3:**
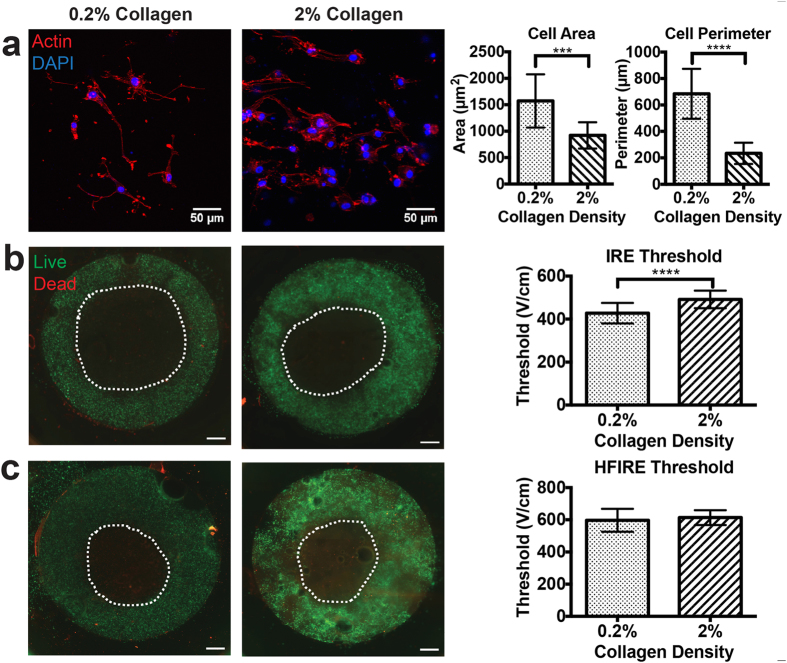
ECM-tuned hydrogels reveal cell size dependent IRE lesions and cell size independent HFIRE lesions. (**a**) Altered cell morphology and overall cell size results from changing density of hydrogel matrix from 0.2% to 2.0% collagen (n = 25, scale bar 50 μm) (**b**) Comparison of IRE treatment for larger cells in 0.2% collagen reveals larger lesion and thus lower death threshold than for smaller cells in 2% collagen (n = 20, p < 0.001) (scale bar 1 mm) (**c**) Comparison of HFIRE treatment in 0.2% and 2% collagen reveals uniform lesions and thus equivalent death thresholds despite cell size differences. (n = 20, p ≥ 0.1) (scale bar 1 mm). (***p ≤ 0.0005 and ****p ≤ 0.0001).

Cell size and shape within hydrogel scaffolds are functions of scaffold density; by varying collagen density in the tissue model we were able to control cell size and outer membrane perimeter for a single cell type. U-87 MG human GBM cells exhibited a significantly smaller area (p = 0.005) in the higher density (2% w/w) collagen (920 ± 249 μm^2^) as compared with lower density (0.2% w/w) collagen (1572 ± 503 μm^2^) (Fig. 3a). Using this *in vitro* model we then determined that these cell geometries determined lethal thresholds for IRE but not for HFIRE pulses. As predicted by the model, IRE lesions for cells in 0.2% collagen were larger than the lesions for cells in 2.0% collagen (Fig. 3b, p < 0.0001). The larger cells were killed by IRE pulses with amplitude exceeding 428 ± 47 V/cm, while the smaller cells required a larger field for cell death (492 ± 41 V/cm). In contrast, HFIRE treatments did not result in statistically significant differences in lesion size, corresponding to an average lethal threshold of 601 ± 65 V/cm that was independent of collagen density (Fig. 3c). The electrical conductivity for the two scaffolds was experimentally comparable, and cell densities were identical in the two conditions.

**Figure 4:**
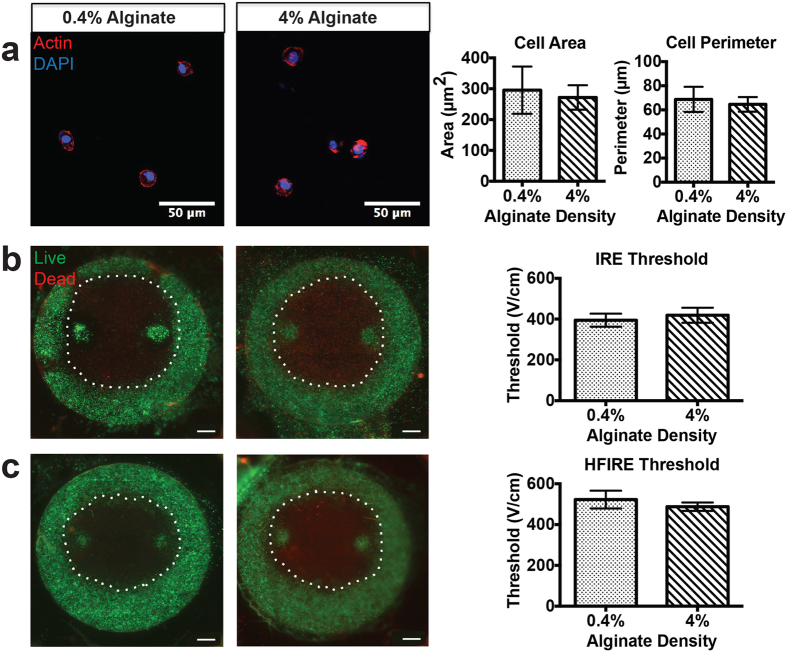
Constant cell morphology with changing stiffness results in equivalent lethal thresholds for IRE and HFIRE. (**a**) Changing the density of alginate does not change cell morphology due to lack of cell-ECM binding sites, allowing for isolating the effect of stiffness on treatments (n = 25) (**b**) IRE lesions and lethal thresholds are equivalent across stiffness differences for equivalent cell morphology (n = 20, p ≥ 0.1) (scale bar 1 mm) (**c**) HFIRE lesions and lethal thresholds are equivalent across alginate stiffness differences (n = 20, p ≥ 0.1) (scale bar 1 mm).

We performed additional experiments in calcium alginate hydrogels, in which cell morphology is relatively constant for different scaffold densities due to the lack of cell-ECM binding sites (Fig. 4a). In alginate hydrogels, lesion sizes and lethal thresholds were independent of polymer concentration for both IRE (Fig. 4b) and HFIRE (Fig. 4c).

### *In vivo* selectivity of IRE

**Figure 5:**
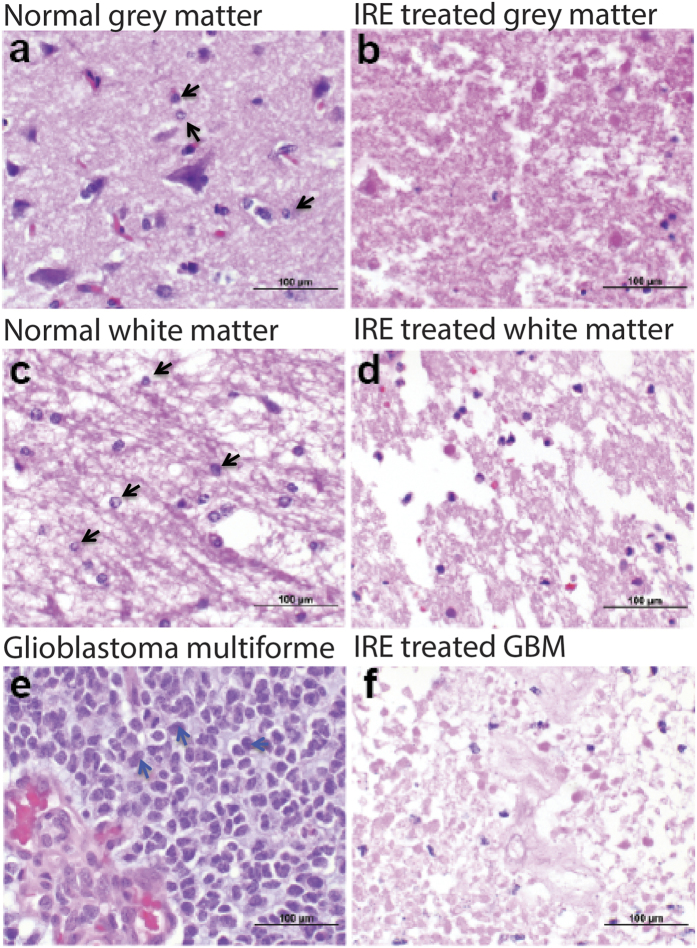
Histomorphology of normal and neoplastic canine brain tissues ablated with IRE. (**a**) Normal, untreated cerebrocortical grey matter (**c**) and white matter of the internal capsule. IRE ablation results in neuronal (**b**) and glial death (**b**,**d**), as well as vacuolization and axonal loss (**d**). Biopsy of glioblastoma multiforme before (**e**) and after (**f**) IRE ablation. The IRE treatment causes disruption of tumor and stromal cytoarchitecture, and tumor cell death. All sections stained with hematoxylin and eosin.

We previously treated canine patients with naturally occurring malignant gliomas using IRE^30^. Histology from this treatment provides an important comparison point between our 3D *in vitro* ablation results presented here, and the *in vivo* outcome in a context that is highly representative of the human GBM phenotype. When untreated cerebrocortical grey matter (Fig. 5a) was exposed to IRE treatment, non-discriminate cell death occurred as both neuronal and glial cells were ablated (Fig. 5b). Similarly, untreated white matter of the internal capsule (Fig. 5c) treated with IRE resulted in glial death in addition to vacuolization and axonal loss. Though malignant glioblastoma cells (Fig. 5e) were ablated with IRE treatment (Fig. 5f), so too is the stromal cytoarchitecture. Based on these *in vivo* results demonstrating the relatively non-selective nature of IRE ablation in canine GBM, combined with our *in vitro* studies demonstrating statistically significant yet small differences in IRE threshold based on cell size, we next focused on the potential for pulsed electric fields to exert cell-specific targeting. Histology images from canine patients illustrate the well-known tumor cell phenotype characterized by the enlarged nuclei of GBM cells (Fig. 5e) compared to healthy tissue (Fig. 5a,c), therefore motivating our hypothesis that intracellular localization of treatment electric fields may enable tumor cell targeting due to nuclear size differences.

### Intracellular effect of pulsed electric fields

**Figure 6:**
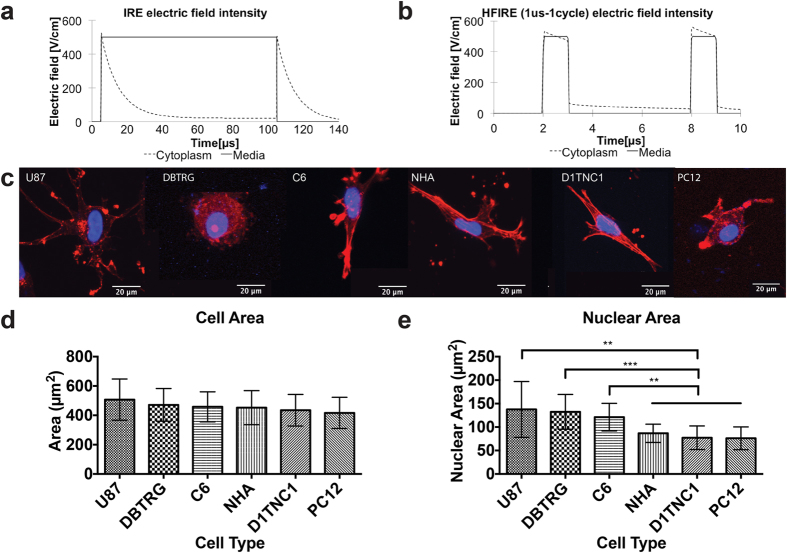
Inner organelle effect of HFIRE predicted to allow for cell-selective differences between malignant and non-malignant cell types by affecting nuclear transmembrane potential. (**a**) Numerical modeling of the electric field produced by IRE pulses predicts the electric field reaches the cytoplasm inside the cell for only a short duration of the pulse time while the majority of the electric field is retained in the media where it aggregates around the cell membrane. (**b**) Numerical modeling of the electric field distribution predicts the electric field produced by HFIRE pulses penetrates through the plasma membrane into the cytoplasm for the entire duration of the pulse on-time. (**c**) Fluorescent imaging of U-87, DBTRG, C6, NHA, D1TNC1, and PC12 cells allows for determination of shape factors to be used in modeling and to correlate to experimental lesion results. (**d**) U-87, DBTRG, C6, NHA, D1TNC1, and PC12 cells show no significant difference (p ≥ 0.1) in overall cell area (n = 20). (**e**) Nuclear area of malignant glioma cells (U-87, DBTRG, and C6) is greater than for non-malignant cells (NHA, D1TNC1, and PC12) (n = 20, **p ≤ 0.005 and ***p ≤ 0.0005).

To examine the potential for HFIRE pulses to exert their effect via intracellular localization of electric fields, we performed finite element modeling of field distribution across a single cell (Fig. S2). This model predicts that for a simulated IRE pulse with an electric field magnitude of 500 V/cm applied for 100 μs, only 14% of the external electric field traverses the cell membrane and is present in the cytoplasm (Fig. 6a). In contrast, HFIRE pulses deliver most of their energy to the inside of the cell (Fig. 6b). The cytoplasm is charged over 400 V/cm for the entire duration of each 1 μs HFIRE pulse while the same is true for only 8% of each 100 μs IRE pulse. To test the implications of effects on tumor cell nuclei for this prediction of a strong intracellular field created by HFIRE, we constructed 3D models using six different cell types (Fig. 6c), chosen to include multiple malignant versus normal cell comparisons. Malignant cell types include two human malignant glioma cell lines (U-87 and DBTRG) and a rat glioblastoma line (C6). Non-malignant cell types include primary normal human astrocytes (NHA), normal rat astrocytes (D1TNC1), and undifferentiated rat neurons (PC12). These 3D cultured cells exhibited no significant difference in cell area (Fig. 6d), but did exhibit significant differences in nuclear area (Fig. 6e). All three tumor cell populations that we cultured exhibited enlarged nuclei when compared with each of the normal cell populations.

**Figure 7:**
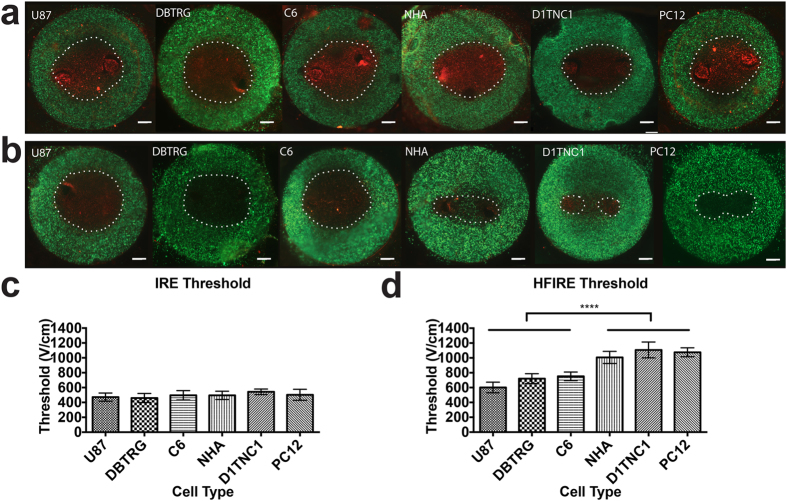
HFIRE threshold is dependent on nuclear size, resulting in cell selective targeting. (**a**) IRE lesion sizes have no significant difference across different cell types (n = 10, p ≥ 0.1). (**b**) HFIRE lesion size for malignant glioma cells (U-87, DBTRG, and C6) is greater than non-malignant astrocytes (NHA and D1TNC1) and neurons (PC12) (n = 10). (**c**) COMSOL modeling relating lesion size to death thresholds shows no significant difference between IRE thresholds for different cell types (n = 10, p ≥ 0.1), confirming the hypothesis that IRE thresholds are primarily dependent on cell size. (**d**) Death thresholds for malignant cells are smaller than normal cells with HFIRE treatment suggesting a range of electric field values that will kill malignant cells without killing healthy cells (n = 10, ****p ≤ 0.0001).

Consistent with model predictions of IRE cell size dependence and nuclear size independence, the four cell types exhibited similar IRE lesions (Fig. 7a). In contrast, HFIRE lesions in the tissue mimics with GBM cells were significantly larger than lesions with non-transformed cell types (Fig. 7b). The similar lethal IRE thresholds across cell types (Fig. 7c) is consistent with the fact that all four cell types have similar outer membrane areas. HFIRE experimental results, however, reveal a lower lethal threshold for malignant cells (Fig. 7d), which have larger nuclei compared with their normal cell counterparts. For HFIRE treatments, U87 human glioblastoma cells were killed at a threshold of 601 ± 71 V/cm, DBTRG human glioblastoma cells were killed at a threshold of 720 ± 67 V/cm, and C6 rat glioblastoma cells were killed at a threshold of 752 ± 58 V/cm. All normal cell types tested had a significantly larger lethal threshold than any of the malignant cell types tested (p < 0.0001). NHAs were killed at a threshold of 1006 ± 81 V/cm, D1TNC1 cells had a lethal threshold of 1107 ± 106 V/cm, and PC12 cells had a lethal threshold of 1076 ± 57 V/cm.

**Figure 8:**
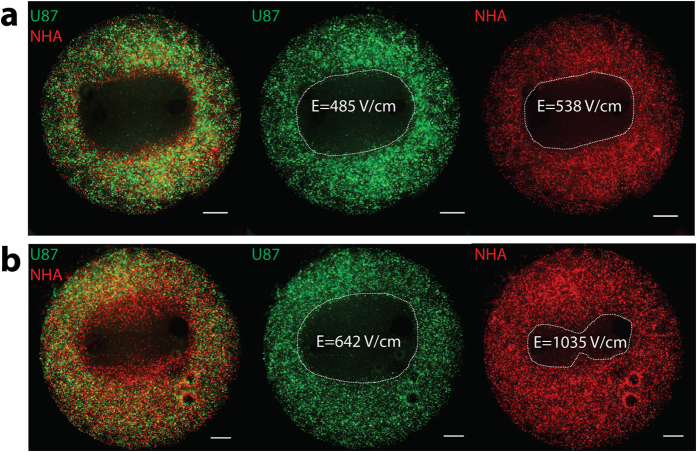
Co-culture treatment demonstrates equivalent lesions with IRE and selective targeting of malignant cells with HFIRE. (**a**) U87 cells (green) and NHA cells (red) co-cultured in a hydrogel and treated with IRE show lethal thresholds in co-culture that match the lethal thresholds seen in monoculture with the lethal threshold of the two cell types being equivalent (scale bar 1 mm). (**b**) U87 cells (green) and NHA cells (red) treated with HFIRE show lethal thresholds in co-culture that match the lethal thresholds seen in monoculture with the lethal threshold of malignant U87 cells being significantly lower than that of the NHA cells resulting in a larger lesion (scale bar 1 mm).

As in monoculture hydrogels, equivalent lesions were achieved for U87 and NHA cells in co-culture hydrogels treated with IRE (Fig. 8a). Selective killing of malignant cells was demonstrated using HFIRE in a co-culture environment, as U87 lesions were significantly larger than NHA lesions within the same hydrogel (Fig. 8b). HFIRE and IRE lethal thresholds for each cell type in co-culture were unchanged from those in monoculture. The selective killing in co-culture demonstrated by HFIRE further supports the feasibility of selective targeting in a complex environment with multiple cell types such as at the periphery of a tumor.

### Death mechanisms of IRE and HFIRE

**Figure 9:**
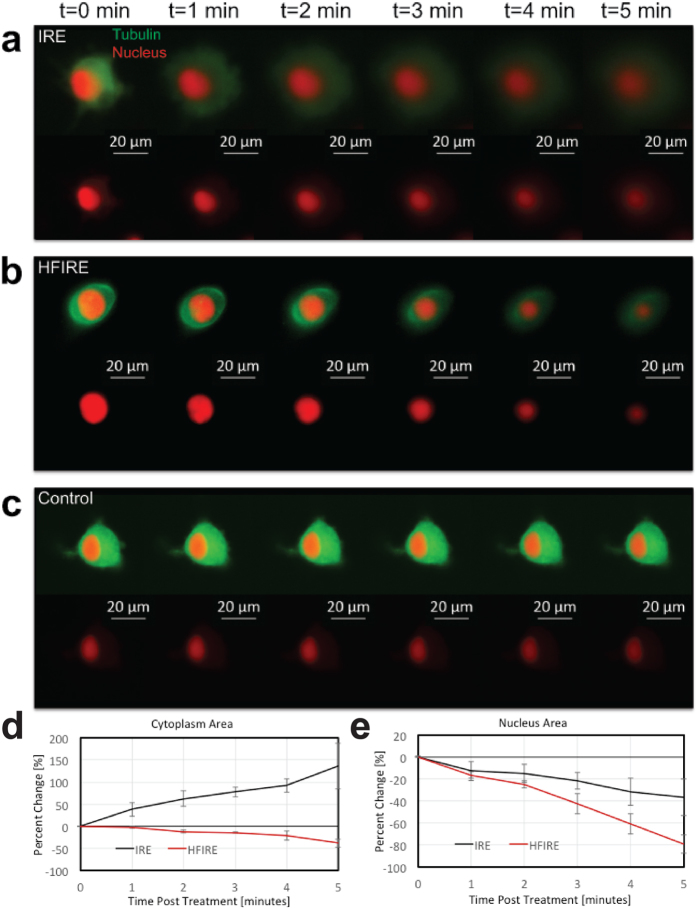
Cell responses after treatment show difference in IRE and HFIRE mechanism. (**a**) Cell exposed to IRE treatment shows a diffusion of stained tubulin from the cell cultured in a 3D hydrogel over a 5-minute time course, suggesting a disruption of the outer cell membrane as a result of pulses. (**b**) Cell exposed to HFIRE treatment shows a sharp collapse of the nucleus, and while tubulin staining dims, it does not clearly diffuse outside of original cell membrane area as in the IRE case. This suggests a different effect on both the nucleus and cell between IRE and HFIRE. (**c**) Cell not exposed to any pulses acts as a control to ensure no photo-bleaching effects from imaging over 5-minute time course. (**d**) Change of cytoplasm area in response to IRE and HFIRE shows a significant difference in the cytoplasmic response to therapy (n = 3, p ≤ 0.0001). Cytoplasm area increases in response to IRE as a result of tubulin diffusion, which is not present with HFIRE. (**e**) Change in nuclear area in response to IRE and HFIRE shows a significant difference in nuclear response to therapy (n = 3, p = 0.0066). The more drastic collapse of the nucleus with HFIRE supports a nuclear effect in HFIRE that isn’t present with IRE.

To investigate the differences between the mechanism of death with IRE and HFIRE we performed single cell imaging upon exposure to each treatment regime. Cell nuclei and tubulin were stained by live fluorescent stain and cultured in 3D collagen hydrogels. Fluorescent imaging *in situ* within these hydrogels was performed directly before, and then at 30-second intervals after exposure to IRE, revealing an outward diffusion of dye from the cell membrane within 1 minute after pulsing (Fig. 9a). By 5 minutes after treatment the tubulin dye had diffused almost entirely out of the cell while the nuclear dye showed a disruption of the integrity of the nucleus. In contrast, cells exposed to HFIRE showed a strong inward collapse of the nucleus followed by a collapse of the tubulin stained cytoplasm on the 5-minute timescale (Fig. 9b). A control cell that was not exposed to either treatment imaged over the same time course confirms that treatment-induced changes are not related to photo-bleaching (Fig. 9c). The marked increase in cytoplasm area with time after IRE treatment is different from the small decrease in cytoplasm area as a result of HFIRE treatment (p < 0.0001) (Fig. 9d). The HFIRE response shows a consistent decrease of the nuclear area with time after treatment that is significantly greater than the decrease in nuclear area evident after IRE treatment (p = 0.0066) (Fig. 9e).

### Estimate of lethal threshold for nuclear disruption

**Figure 10:**
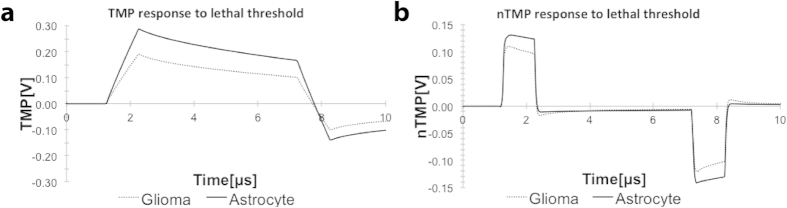
Predicted TMP and nTMP response to HFIRE experimental lethal thresholds for modeled glioma and astrocyte cells suggests a nTMP effect. (**a**) Modeled cells with experimental geometries for glioma cell and astrocytes exposed to simulated HFIRE experimental lethal electric field thresholds for the given cell type show a difference in TMP increase in response. (**b**) Modeled cells with experimental geometries for glioma cell and astrocytes exposed to simulated HFIRE experimental lethal electric field thresholds for the given cell type show a similar nTMP increase in response, suggesting a value for nTMP increase that will cause cell death. TMPs and nTMPs presented in this figure correspond to the surface average.

We next further explored the relationship between HFIRE lethal thresholds and nuclear size, leveraging our experimental data as input for subsequent mathematical models. We hypothesized that cell death occurs at a critical nTMP disruption that is independent of cell type, whereas the external field required for this nuclear disruption scales inversely with nucleus size. Using experimental findings for lethal thresholds, nuclear geometries, and idealized cell geometries for glioma cells and astrocytes, we performed finite element modeling of single cell response to minimum lethal electric fields for each cell type. In search for the potential mechanism of action for HFIRE, we simulated electric field magnitudes of 1006 V/cm for NHA cells and 601 V/cm for U-87 cells. We found a larger increase in TMP for the astrocyte than for the glioma cell (Fig. 10a), however these TMPs were significantly below the anticipated 1 V instantaneous lethal threshold for IRE. In contrast, simulation of nTMP response across the entire area of the nuclear envelope predicts similar increases in nTMP for both cell types, indicating that irreversible electroporation is occurring at a common value (~130 mV) of nTMP for both cells (Fig. 10b).

## Discussion

The overall goal of our current study was to leverage tissue engineered models of tumor versus normal brain microenvironments, based on our previously published methods^31^, to investigate the response of representative cell geometries to IRE and HFIRE pulses. These platforms critically provide a three-dimensional physiological tissue context in which to explore the effects of 3D cell morphology on response to electric fields, not possible with 2D experiments, while eliminating other confounding variables found *in vivo*. Hydrogels have been previously established as a relevant platform to test tissue responses to IRE pulses^19^, while such models have also been demonstrated to better recapitulate human tumor physiology and therapy response as compared with 2D models^32^,^33^. With the ability to easily tune targeting parameters and microenvironment, these models provide a valuable tool for measuring the impact of cell morphology and tissue physics on therapy response broadly, and more specifically on response to therapeutic electric fields, which are the focus of this study.

It is important to note that our work is informed by, and builds on our experience in treating spontaneous GBM in canine patients. Spontaneous, primary brain tumors are only relatively common in two species – dogs and humans. Human and canine brain tumors share many features, including histopathologic and diagnostic imaging characteristics, which allows application of World Health Organization pathologic classification and imaging based therapeutic response assessment schemes used in human clinical practice. Canine and human brain tumors have also been demonstrated to have similar expression patterns of growth factor receptors, chromosomal deletions, and losses of function of tumor suppressor genes. As tumors progress 5- to 7-fold faster in dogs relative to humans, dogs with spontaneous brain tumors are an attractive model for the faithful and rapid evaluation and translation of novel brain tumor therapeutics^34^. While our *in vivo* work in treating primary canine GBM tumors demonstrates the great potential for IRE for the treatment of human GBM, this work also demonstrates a limitation inherent in IRE in terms of its potential for tumor cell specific ablation. However our 3D models are significantly more amenable to exploring cell-level responses and death mechanisms, needed to advance electric field ablation to a more cell-targeted modality.

Size selective ablation using PEFs has been previously reported, both on the single cell level in 2D culture^35^, and in cell suspensions for the application of differentiating tumor from blood cells based on large differences in size^36^, but has yet to be demonstrated for cells cultured in physiologically-relevant tissues. Our experiments support the concept that IRE results in cell size-selective lethal thresholds in 3D tissues. The bulk electrical resistance properties of the cell-seeded hydrogels did not vary as a function of collagen density, and we therefore believe differences measured are a result of cell morphology rather than altered tissue electrical properties. Control experiments performed in alginate further support this hypothesis that the differences observed in collagen resulted from cell size variations rather than additional factors such as direct sensing of matrix density. Although this finding does not eliminate the possibility that variables such as variation in binding ligand density, matrix structure, and matrix mechanics may also impact lesion size, this size dependence is consistent with previously published data on cells in solution^36^. Furthermore this correlation of threshold with altered matrix density is absent for HFIRE, suggesting a cellular effect rather than a matrix effect. We hypothesize that this is due to the HFIRE field primarily interacting with the inner organelles of the cell. There is the possibility that the IRE changes are related to cell-ECM interaction changes that are sensed for IRE response, which effects the outer membrane, but not HFIRE, which is hypothesized to be enhanced by differences in intracellular structures. However, because the effect here is so small over a large ECM density range, we don’t believe this will impact therapy. Our finite element modeling supports the hypothesis that HFIRE can induce intracellular effects. A single HFIRE burst applied to a single cell model produces a much higher field inside the cell than a simulated IRE burst. HFIRE treatment delivers a rapid burst of over 100 of these 1 μs pulses (Fig. S3). This allows HFIRE pulses to preferentially charge intracellular membranes, which we anticipated would have profound effects on cell death as a function of cell type.

Our *in vitro* 3D model results demonstrate a statistically significant dependence of field threshold on cell size, however the narrow range of selectivity evident along with the degree of cell size heterogeneity observed *in vivo* may prevent this dependence from being leveraged for targeting specificity. A much more obvious difference between cell types, clearly evident in our H&E staining of tumorous and healthy canine brain samples, is the enlarged nuclei of cancer cells compared to healthy brain tissue. Therefore, enlarged nuclei of cancerous cells represent the metric we explored for selective targeting. Used as a pathological indicator of cancer, enlarged nuclei compared with their non-malignant counterparts are one of the most reliable distinguishing characteristics of tumor cells^37^, however the targeting of anti-cancer therapy against this hallmark has never been demonstrated. We justified our assumption in using enlarged nuclei as a predictor of malignancy because it is a fundamental morphologic marker for cancer, and is nearly universally applied when diagnosing cancers, including brain tumors. Although it is not as important in brain malignancies compared to some other cancers, nuclear pleomorphism is a criteria used when grading brain tumors as described by the WHO^38^. The exuberant giant, multinucleated features present in some glioblastomas provide an excellent example of the extreme nuclear pleomorphism that can be present in cancer. It is true that histologic and other morphologic criteria are gradually being supplemented and improved when complemented with genetic and other diagnostic criterion towards the development of personalized medicine. However, as the WHO classification system is currently an accepted and the most widely used gold standard, and histological diagnosis remains a primary method of cancer diagnosis, the nuclear pleomorphism and NCR are still a valid surrogate of malignancy.

The nucleus is typically the largest contiguous intracellular feature and a likely target for damage by the high intracellular fields produced by HFIRE. To experimentally test the effect of nuclear area on treatments, we chose different cell types, which exhibited differences in nuclear sizes without significant differences in plasma membrane area, allowing us to eliminate confounding effects due to cell size. Numerical simulations identified increased nuclear size as an important variable for increased nTMP. We hypothesized that an increase in nTMP could trigger cell death above a specific threshold, and therefore malignant cells with enlarged nuclei should have a lower HFIRE lethal threshold than normal cells, in contrast with IRE, which would not exhibit nuclear selectivity. The similarity of IRE thresholds is consistent with the fact that there was no significant difference in plasma membrane areas. The differences in HFIRE lesion sizes supports the hypothesis that HFIRE threshold differences are related to nucleus area as opposed to overall cell area, with lower lethal thresholds corresponding to larger nuclei. The intracellular field produced from HFIRE seems to affect the nucleic membrane in a way at least partially analogous to the way IRE affects the plasma membrane, as a larger membrane exposed to the majority of the electric field is easier to affect than a smaller membrane. Our main goal in this work is to demonstrate that enlarged nuclei may provide a viable target for HFIRE therapies. A critical point for the purpose of this study is that the cell populations we have chosen exhibit significant differences in the morphological features we are interested in, namely enlarged nuclei. Here we show a relationship between enlarged nuclei and lower lethal thresholds with HFIRE treatment, which we believe we will be able to leverage in future work *in vivo* due to the known enlargement of NCR in cancer. It may be possible to sort malignant populations based on marker expression and to then establish a robust understanding of the connection between HFIRE lethal thresholds and molecular signatures. Our continuing work will follow this important line of study, specifically involving the HFIRE response of glioma cells expressing differing levels of known cancer stem cells markers.

By varying collagen matrix density we show that GBM cells in 0.2% collagen demonstrate a more elongated morphology than in 2% collagen, which may be similar to the morphology changes seen in invasive cells, which tend to elongate and lose bulk in the cytoplasm surrounding the nucleus. However, no experimental steps were taken to specifically induce an invasive phenotype in these cells. Based on histological examination of invasive cells, it appears that an enlarged nucleus in malignant cells is conserved even during the dynamic process of invasion. Though further investigation is necessary, the results presented here indicate that HFIRE should be capable of targeting these invasive cells.

Time-course images of single cells exposed to each treatment show a distinct difference in mechanism of killing between HFIRE and IRE, consistent with the findings that different cellular characteristics are important variables with the two treatments. The time-course of cell death after IRE treatment strongly implicates the immediate disruption of the cell membrane as a cause of cell death, as tubulin proteins originally confined in the cell by the cell membrane begin diffusing out of the cell upon exposure to IRE. In contrast, cells exposed to HFIRE show no diffusion from the outer cell membrane but rather a nuclear collapse while the tubulin is retained within the original cell area. These findings suggest that while the outer membrane may be subject to a small degree of electroporation, it does not play as much of a role in the mechanism of cell death in HFIRE, but rather that the primary effect is on the nucleus.

Given our results, we hypothesize that HFIRE is acting on the biophysical structure of the cells in a way that nuclear area becomes a key variable. When glioma and astrocyte cells were simulated at their respective lethal HFIRE thresholds (601 V/cm vs. 1006 V/cm), we found similar TMP and nTMP ranges of approximately 150–250 mV and 100–130 mV, respectively. These simulations did interestingly predict a small difference in outer TMP as a function of nuclear size. However the magnitude of this TMP, approximately 150 mV, was significantly lower than the anticipated instantaneous threshold (1 V) for cell death by irreversible electroporation. This supports the hypothesis that the primary mechanism of death with HFIRE is not an increase in cell TMP, but rather is related to intracellular effects. For glioma and astrocyte cells, the maximum simulated nTMP of 130 mV is also well below the lethal threshold for death resulting from outer membrane disruption, suggesting that small disruptions of nTMP may significantly impact cell survival. It is unclear whether the pathway to cell death is dominated by effects on the nuclear envelope alone, versus in combination with cell membrane disruption, or a separate cascade of intracellular effects. However, the correlation of nTMP values between the two different cell types, at different lethal electric field strengths, indicates that nuclear area impacts the cell death process after HFIRE treatment.

Our mathematical model does have limitations, as outer cell membranes are approximated as elliptical, and do not account for the irregular shape of physiological cells, or heterogeneity in electrical properties of individual cells. Inclusion of membrane conductivity changes due to electroporation effects would also enhance the accuracy of our simulations. While IRE models accounting for such effects do exist, these have not been characterized for HFIRE pulses. Characterization of the cell membrane response to HFIRE pulses (e.g., conductivity, porosity) is beyond the scope of this project thus both models are presented with non-dynamic cell membrane properties. As pulse-width of HFIRE pulses increases TMP values start approaching those of IRE. It is important to note that the cell-specific thresholds presented in this paper may no longer hold for different pulse widths.

While experimental evidence also suggests that outer membrane electroporation is occurring during HFIRE (at time-points beyond those in Fig. 8, data not shown), our experimental results and model findings strongly suggest an active role for nTMP effects in the HFIRE mechanism of action. It is widely recognized that the mechanism of death in irreversible electroporation using short pulses is complex, poorly understood, and can follow multiple different pathways^22^. Furthermore, nuclear poration may be aided not only by increased nuclear size of cancer cells but also other abnormalities of the nucleus such as reduced nucleus stiffness necessary for invasion^39^. Another possibility is an amplification of the electric field applied to the cytoplasm caused by distortion around an enlarged nucleus. This may result in other inner organelles, such as mitochondria, being disrupted by HFIRE pulses. Future work will be needed to explore these additional effects, however our results highlight the importance of TMP increases in both IRE and HFIRE and nTMP increases specifically associated with HFIRE, in determining cell death PEF thresholds.

It is important to note, the electric field therapies explored in these studies differ from the alternating electric field treatments being used clinically^40^. These tumor treating fields (TTFields), such as the Optune^TM^ system (Novocure, Saint Helier, Jersey), have specific inhibitory effects on dividing cells, while HFIRE and IRE target the physical properties of cells through membrane disruption. While having the benefit of being less invasive than HFIRE treatment, TTFields rely on targeting the properties of highly proliferative cells, and would leave behind the quiescent tumor initiating cells that cause recurrence. Because IRE and HFIRE operate via a different mechanism, they should elicit a death response through membrane disruption for both bulk tumor cells and non-dividing tumor initiating cells. In addition, it is unlikely that this physical death mechanism results in the emergence of resistant subpopulations on short timescales, because a large number of genetic mutations would likely be required to render a cell resistant to electric field-induced damage.

Because an enlarged nucleus is a conserved phenotype in malignant cells and HFIRE is not dependent on cell size, it is hypothesized that consistent and tunable lesions can be achieved in heterogeneous tumors so as not to leave behind cells that will repopulate the tumor. There is certainly heterogeneity in nuclear size of cells in malignant tumors and therefore HFIRE will not be perfectly able to selectively kill all malignant cells. However, when HFIRE is used as a supplement to current therapies, any cells left behind from HFIRE due to nuclear area heterogeneity are unlikely to also be resistant to the adjunctive therapies as these therapies operate by different mechanisms. Because malignancy correlates with altered nuclear morphology, the malignant selection mechanisms should be different with this method than other treatment methods and should not leave behind highly malignant cells. A major difference between HFIRE and other therapies that select out resistant populations is that HFIRE acts on physical aspects of the cell, which are highly conserved in malignant populations. Based on our results, we also show HFIRE selectivity may be beneficial because there is no associated dose limiting toxicity (DLT) to normal tissues. DLT, biological resistance/escape, and off target effects are major problems associated with chemotherapy, radiotherapy, and molecular immunotherapies, which may not be an issue with a treatment based on physical properties of cells. However, all these hypotheses will need to be tested in more complex models of disease.

It is important to note that the results reported here were obtained in an *in vitro* model of disease, which was intended to maximally replicate *in vivo* morphological features, by tuning matrix conditions, while also minimizing confounding factors. It is likely that local and systemic immune effects will be observed when this therapy is implemented *in vivo.* It is unclear if a differential immune effect between HFIRE and IRE treatments will be observable due to the relative intracellular and membrane targeting processes and future studies in appropriate *in vivo* models of disease will be necessary to optimize protocols which result in targeting of malignant cells.

Though the exact mechanism of cell killing with HFIRE is not yet known, our modeling and experimental data suggest a mechanism that is different than that of long IRE pulses which target the plasma membrane, and that, unlike for IRE, is cell type dependent among cells of similar size. The HFIRE killing mechanism is such that the biophysical structure of malignant cells allows for the selective targeting of these cells using a range of electric field distributions that induce no damage to the healthy cells studied but elicit a death response in malignant cells. Though it is unlikely that 100% selective killing of malignant cells can be achieved due to the heterogeneity that does exist in physical properties of cells, HFIRE can be used to ablate all cells within the tumor margin and pulse parameters can be tuned to achieve preferential killing of a significant fraction of the malignant cells past the tumor margin. Because malignant cells that comprise the tumor have a lower death threshold (~530–810 V/cm) than normal astrocytes (~930–1200 V/cm) surrounding the tumor, it follows that a treatment regime delivering a voltage between these two thresholds to the edge of the tumor may result in ablation of tumor cells while sparing healthy astrocytes. While the response of other cell types and structures within the brain parenchyma must be investigated in future work, a threshold in such a range at the edge of the tumor may be effective at killing the invasive glioblastoma cells that render surgery to be an ineffective treatment for GBM, and infiltrative tumors more broadly.

## Materials and Methods

### Cell culture

U-87 MG primary human glioblastoma cells (ATCC), D1TNC1 rat astrocyte cells (ATCC), and C6 rat glioblastoma cells (ATCC) were all cultured in Dulbecco’s Modified Eagle Medium (DMEM) containing 10% fetal bovine serum (FBS) and 1% penicillin/streptomycin (PS). Normal Human Astrocyte (NHA) cells (Lonza) were cultured in Astrocyte Growth Media (Lonza). PC12 undifferentiated rat neurons (ATCC) were cultured in DMEM containing 5% horse serum, 5% calf serum and 1% PS. DBTRG human glioblastoma cells (ATCC) were culture in RPMI medium containing 10% FBS, 2 mM L-glutamine, 1% PS and 0.1 mM non-essential amino acids. All cells were grown in culture at 37 °C in 5% CO_2_ in a humidified incubator. Cells were seeded in hydrogels at a density of 1 × 10^6^ cells/mL. The hydrogels were submerged in appropriate growth media for the cell type at 37 °C in 5% CO_2_ in a humidified incubator and cell viability was maintained within hydrogels for up to 7 days (Fig. 2a).

### Construction of 3D collagen scaffolds

Stocks of type I collagen were prepared by dissolving rat tail tendon in acetic acid, followed by freezing and lyophilization as described previously^19^. Two different stock solution concentrations of collagen were created: 4.5 mg/mL and 30 mg/mL. Scaffolds with a final concentration of 2 mg/mL and 20 mg/mL were made from concentrated collagen stocks to create collagen gels of 0.2% (w/w) and 2% (w/w). Neutralized collagen solutions were created by mixing acid-dissolved collagen with 10X DMEM (10% of total collagen solution volume) and sufficient volumes of 1N NaOH until a pH in the range of 7.0–7.4 was achieved. The neutralized collagen was mixed with cells suspended in DMEM or NHA media to achieve a cell density of 1 × 10^6^ cells/mL in the final collagen mixture. Solutions were mixed carefully with a spatula to ensure homogenous distribution throughout the gel without damaging cells. Collagen solutions were then dispensed into a polydimethylsiloxane (PDMS) mold with a cut-out of 10 mm diameter and 1 mm depth and molded flat to ensure consistent scaffold geometry (Fig. S1). Our previous mathematical modeling and experiments on oxygen (O_2_) consumption rates by tumor cells^31^ confirms that at this cell density and scaffold thickness, O_2_ concentration is uniform throughout the scaffold depth. Collagen was allowed to polymerize at 37 °C and 5% CO_2_ for 30 minutes.

### Construction of 3D alginate scaffolds

Calcium alginate gels were created using the same PDMS molds as for collagen, creating discs 10 mm in diameter and 1 mm in thickness. Two alginate gel stock concentrations (0.4% and 4.0% (w/v) were prepared using powdered alginate (Protanal LF 10/60, FMC BioPolymer) that was dissolved in buffer, dialyzed, frozen and lyophilized, followed by re-constitution in serum-free DMEM, as we have previously reported^31^. Alginate concentrations were chosen to span a wide range in mechanical stiffness, similar to the collagen concentrations used. Alginate solutions were mixed with cells at a density of 1 × 10^6^ cells/mL and dispensed into PDMS molds and molded flat with a porous membrane. Alginate hydrogels were cross-linked by submerging under 0.1 M CaCl_2_ dispensed over a porous membrane cover for 45 min. The alginate hydrogels were then cultured in 24 well plates with DMEM supplemented with 10% FBS and 1% PS at 37 °C, 5% CO_2_.

### Construction of co-culture scaffolds

Before seeding cells into collagen hydrogels, U87 cells were incubated for 30 minutes with calcein green, AM (Molecular Probes, Eugene, OR) and NHA cells were incubated for 30 minutes with calcein red-orange, AM (Molecular Probes, Eugene, OR) to distinguish cell populations from each other in co-culture. After staining, cells were rinsed and seeded into collagen hydrogels at a totally density of 1 × 10^6^ cells/mL of collagen with each cell type making up half of the total cell density. Electroporation treatment was delivered 12 hours after seeding cells into collagen scaffolds. Upon electroporation treatment, calcein stains were no longer fluorescent in dead cells and lesion diameters were measured from the cells that were fluorescent 1 hour after treatment.

### Determination of shape factors

U-87, DBTRG, C6, NHA, D1TNC1, and PC12 cells were individually seeded in hydrogels of one of the four conditions described previously (0.2%, 2% collagen, 0.4%, 4% alginate). After culturing the cells for 24 hours, the hydrogels were fixed using 4% formalin and blocked and permeabilized using 40 mg/mL bovine serum albumin (BSA) and 0.05% Triton-X. Cellular actin was stained with Alexa Flour 568 phalloidin (Life Technologies, Carlsbad, CA) while cell nuclei were stained with diaminophenylindole (DAPI; Sigma-Aldrich, St. Louis, MO). Cells were visualized using a Zeiss LSM510 (Carl Zeiss Microscopy LLC, Thornwood, NY) laser scanning confocal microscope. The stained cells were then used to determine cellular shape factors for cells in each of the four conditions. Image analysis was done in Image J (NIH, Bethesda, MD) to determine the nuclear area, nuclear perimeter, cytoplasmic area, cytoplasmic perimeter, and longest and shortest diameter of the cell. Z-stack images were converted into 2D projection images and cell measurements were made from these projections. Measurements were made on at least four cells per hydrogel and at least 5 hydrogels were analyzed for each condition.

### Live fluorescent imaging

U-87 cells were cultured under normal culture conditions and incubated for 16 hours with CellLight Nucleus-RFP, Bacman 2.0 (Molecular Probes, Eugene, OR) and CellLight Tubulin-GFP (Molecular Probes, Eugene, OR) added to the media at a concentration of 10 particles per cell. Cells were then passaged and seeded into hydrogels of a final concentration of 0.2% collagen at a density of 1 × 10^6^ cells/mL. After cells were cultured in collagen hydrogels for 24 hours, electroporation of hydrogels was performed on the stage of a Zeiss Observer Z1 microscope (Carl Zeiss Microscopy LLC, Thornwood, NY) to allow for imaging during treatment. Images were taken of single cells immediately before pulsing treatments were started and then every 30 seconds for 5 minutes after pulsing began. Cells were imaged upon exposure to IRE treatment or HFIRE treatment. Cells that were not exposed to pulses were also imaged as a control.

### Electroporation of 3D scaffolds

Pulsed electroporation experiments were performed in hydrogels with constant electrical properties. The electrical conductivities of each of the gel-cell mixtures were measured with a conductivity meter to ensure similar electrical properties (0.98 ± 0.04 S/m). The IRE pulses were generated using an ECM 830 pulse generator (Harvard apparatus, Holliston, MA) and delivered to the tissue through custom electrodes. High- frequency pulses were delivered using a custom-built pulse generation system (INSPIRE 2.0, VoltMed Inc., Blacksburg, VA). Two solid stainless steel cylinders with diameters of 0.87 mm, separated 3.3 mm edge-to-edge, were used as electrodes.

Treatments were performed delivering a total of 50 square pulses (IRE) or 50 bursts of 1 μs pulses (HFIRE). The IRE protocol delivered 100 μs pulses with a repetition rate of 1 pulse per second. In the HFIRE protocol, a burst consisting of 100 × 1 μs pulses with a 5 μs inter-pulse delay was delivered as shown in Fig. S3 with a repetition rate of 1 burst per second. For IRE treatments, the pulse amplitude was set to 450 V_peak_ while for HFIRE treatments 700 V_peak_ was used to produce ablations of approximately the same volume as the IRE group.

### Finite element analysis in hydrogels

Finite element models using COMSOL Multiphysics (Version 4.3, COMSOL Inc., Palo Alto, CA) were used to solve the Laplace equation to find the electric field distribution within the hydrogels for each different voltage used (Supplemental Note 1). COMSOL Multiphysics was also used to solve the Joule heating equation to calculate the temperature distribution in the hydrogel as a result of each treatment (Supplemental Note 2). The simulation geometry was modeled as a 10 mm diameter and 1 mm thick cylinder with two steel electrode cylinders (d = 0.87 mm) spanning the depth of the hydrogel. Thermal and electrical properties for each domain can be found in Table S1. The mesh was refined until error between successive refinements was less than 1%. The final mesh contained 47,438 elements and solutions were found in approximately 3 minutes on a Pentium i3 processor.

### Finite element analysis of individual cells

The transmembrane potentials across the cell membrane and nuclear envelope were modeled using a finite element model with an impedance boundary condition scheme^24^. These finite element models were used to numerically investigate the response of representative cell geometries to simulated IRE and HFIRE pulses. Cell geometry was determined based on average measurements made in ImageJ image analysis software (NIH, Bethesda, MD) from confocal microscopy images. Geometries for U-87 cells in two different collagen densities (0.2%, 2%) as well as four different cell types (U-87, NHA, C6, D1TNC1) in a 0.2% collagen matrix were used. All models were solved using a 2D-axisymmetric platform in COMSOL Multiphysics. A separate electric currents physics module was used for each domain (media, cytoplasm, nucleoplasm) (Fig. S2). A large media domain, with sides of 300 μm, was used to avoid any significant boundary effects. The cell and the nucleus were modeled as half-ovals where their lengths and widths were varied according to measurements from confocal microscopy images (Table S2).

Simulations were solved in the time-domain using an electric currents module. To account for the resistance and capacitance posed by the cell membrane and the nuclear envelope the boundaries of the nucleus and cytoplasm were assigned impedance properties based on the existing literature, as summarized in Table S2.

### Determination of lethal thresholds

The thresholds for cell death were determined by first performing a live-dead stain on the hydrogels 24 hours after delivering treatment. Live cells were stained with Calcein AM (Biotium, Hayward, CA) and fluoresced as green while dead cells were stained with ethidium homodimer III (Biotium, Hayward, CA) and fluoresced as red. The diameter of the red-stained dead region was measured using ImageJ image analysis software. Geometric measurements of the ablation zones were mapped to a finite element model to calculate the electric field during treatments of the scaffolds (Fig. 2c). The electric field magnitude at the edge of the live and dead regions was considered the electric field threshold for cell death for the given cell type. Imaging of samples presented some background noise mainly due to debris from the remaining 3D microenvironment and re-seeding of detached cells post-treatment.

### *In vivo* canine treatment

All canine *in vivo* studies were approved by the institutional animal care and use committee (08-218-CVM). All methods were carried out in accordance with the approved guidelines. IRE treatments were performed in the brains of anesthetized normal canine subjects, and in dogs with spontaneous malignant gliomas according to previously described methods^21^,^30^,^41^. In tumor-bearing dogs, biopsy of the brain lesion was performed prior to IRE ablation to allow for histopathological diagnosis and grading of tumors, and an additional biopsy of the ablated region obtained within 24 hours of the IRE to characterize the effects of the IRE treatment.

### Histomorphological staining

Archived, paraffin embedded, transversely oriented brain sections from normal and tumor-bearing dogs treated with IRE were retrieved, cut at 5 μm thickness, mounted on positively charged slides, and stained routinely with hematoxylin and eosin^21^,^41^. Digital photomicrographs of regions of interest representing IRE ablated regions of cerebral cortex, subcortical white matter, contralateral homologous cortical and white matter controls, and a canine GBM pre- and post-IRE treatment were captured with charge-coupled device digital camera (Nikon DS-Fi1c, Nikon, Japan) and commercial imaging analysis software system (NIS Elements AR, Nikon, Japan).

### Statistical Analysis

Statistical significance was determined by a two-tailed *t-*test performed in Prism Statistical Software (Version 6, Graphpad, La Jolla, CA). A 95% confidence interval was used with significance defined as p < 0.05. All numerical results are reported as the mean and the standard deviation of all experimental measurements. No outliers were excluded.

## Additional Information

**How to cite this article**: Ivey, J. W. *et al.* Targeted cellular ablation based on the morphology of malignant cells. *Sci. Rep.* **5**, 17157; doi: 10.1038/srep17157 (2015).

## Supplementary Material

Supplementary Information
