# Supplementary material for: Targeted cellular ablation based on the morphology of malignant cells
Source: Sci Rep. 2015 Nov 24;5:17157. doi: 10.1038/srep17157 (PMC4657158; doi:10.1038/srep17157)
Supplement: Supplementary Information [file srep17157-s1.pdf]

Supplementary information

Targeted cellular ablation based on the morphology of malignant cells

Jill W. Ivey[1†], Eduardo L. Latouche[1†], Michael B. Sano[1,2], John H. Rossmeisl[3,4], Rafael V. Davalos[1,4*], Scott S. Verbridge[1,4*]

[1]Department of Biomedical Engineering and Mechanics, Virginia Tech-Wake Forest University, Blacksburg, VA, 24061

[2]Department of Radiation Oncology, Division of Radiation Physics, Stanford University School of Medicine, Stanford, CA 94305

[3]Neurology and Neurosurgery, Virginia-Maryland College of Veterinary Medicine, Blacksburg, VA, 24061

[4]Comprehensive Cancer Center, Wake Forest Baptist Medical Center, Winston-Salem, NC, 27157

**Corresponding Authors: davalos@vt.edu, sverb@vt.edu*

[†]These authors contributed equally to this work

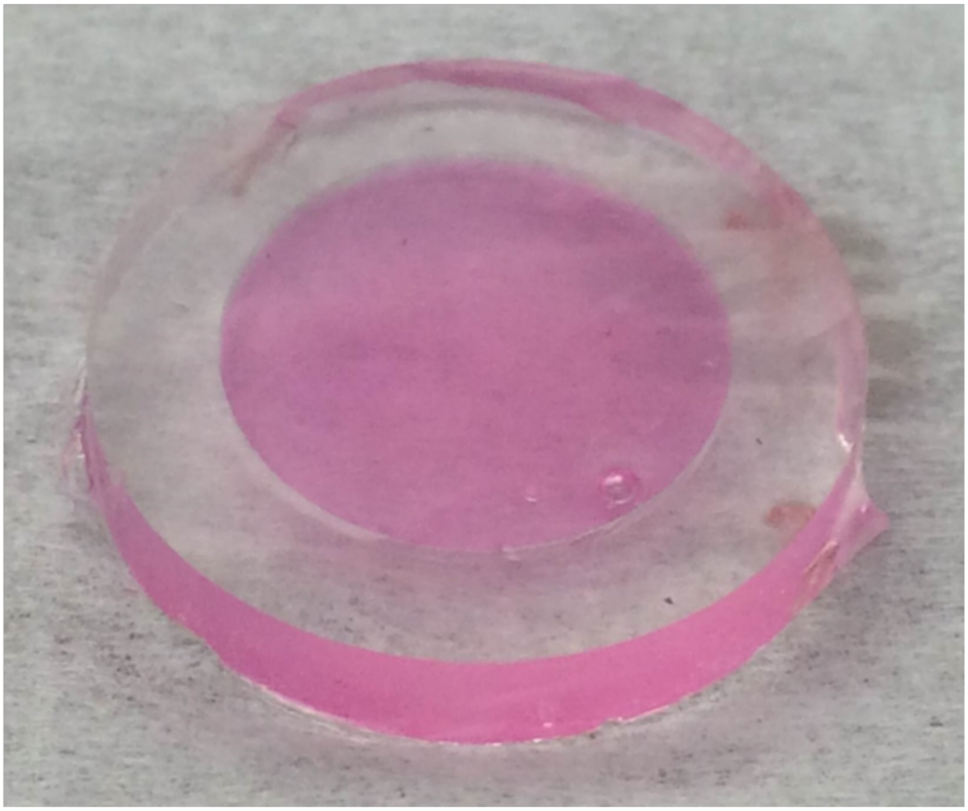

Supplementary Figure 1. Photograph of cell-seeded collagen hydrogel maintained in PDMS well.

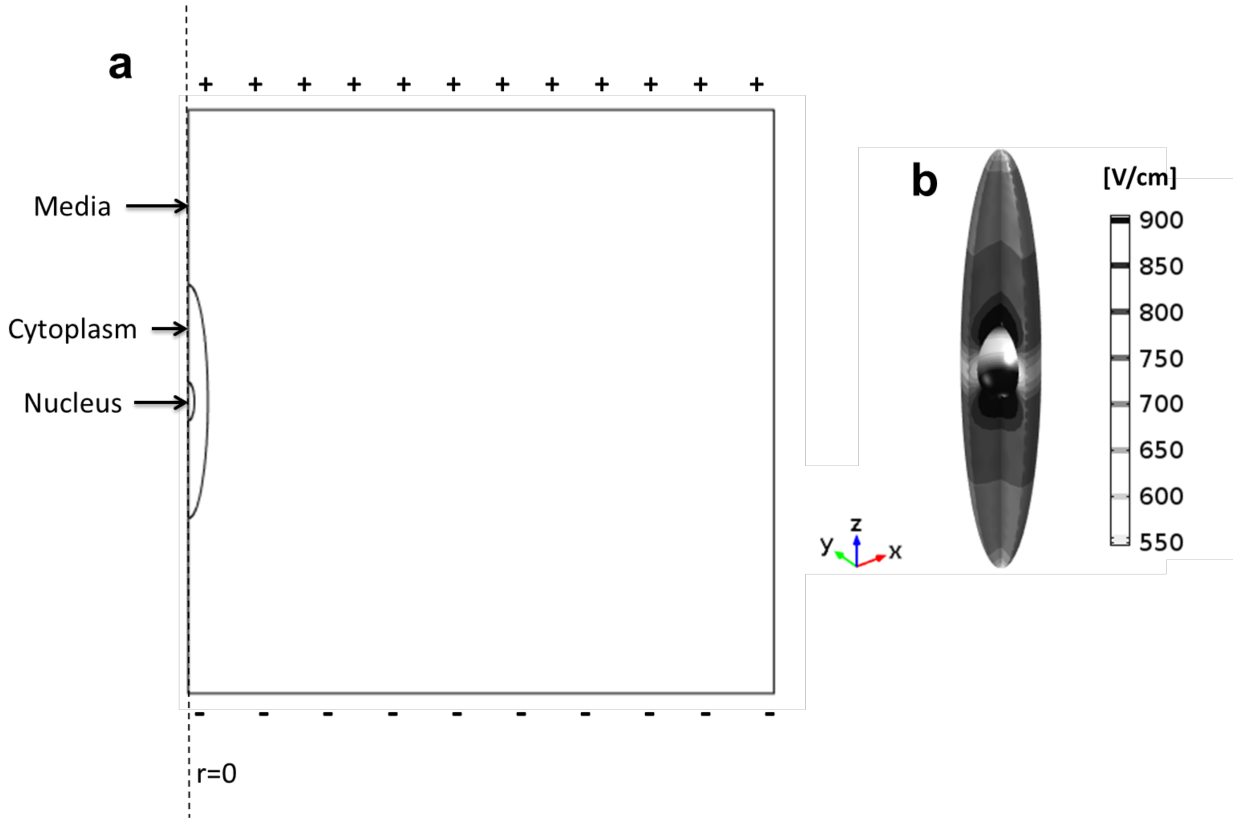

Supplementary Figure 2: Individual cells modeled in a large domain 2D axisymmetric domain. (**a**) For numerical modeling of a single cell an electric field is applied across a block of media and a cell centered within. Changes in TMP and nTMP are calculated from time-dependent solutions. (**b**) Electric field isocontours in cytoplasm for HFIRE therapy (700V) of glioma cell model

For most single cell simulations the final mesh contained around 3,000 elements and solutions were found in no longer than 1 minute on a Pentium i3 processor. For each domain (media, cytoplasm, nucleoplasm), a separate Electric Currents physics module was used and the dependent electric potential variables ϕ_{media},ϕ_{cyto}, ϕ_{nuc} for the media, cytoplasm, and nucleoplasm domains were defined, respectively. These variables were then defined to calculate the voltage across the cell membrane (ϕ_m) and nuclear envelope (ϕ_n)

$$\phi_m = \phi_{media} - \phi_{cyto} \qquad (1)$$

$$\phi_n = \phi_{cyto} - \phi_{nuc} \qquad (2)$$

In each Electric Currents module, the boundaries representing membranes were defined as impedance boundary conditions with reference potentials prescribed as the electric potential in the adjacent (φ_{ref}) domain

$$\boldsymbol{n}\cdot(\boldsymbol{J}_1-\boldsymbol{J}_2)=\frac{1}{d}\left(\sigma(\phi-\phi_{ref})+\varepsilon_0\varepsilon_m\frac{\partial}{\partial t}(\phi-\phi_{ref})\right) \tag{3}$$

where σ is the conductivity, ε_0 is the permittivity of free space, ε_m is the relative permittivity, and d is the thickness of the cell membrane or nuclear envelope. The boundary was defined as a 'thin layer' and the electrical conductivity, relative permittivity, and surface thickness were defined using the values presented in Supplementary Table 2. Domain boundaries perpendicular to the z-axis (z = ±150 μm) were defined as time domain voltages (ϕ = V(t)) simulating the 1 or 100 μs pulses with rise times of 16 ns or 10 μs, respectively. The remaining external boundary was given electrically insulating properties ($-\boldsymbol{n}\cdot\boldsymbol{J}=0$). To simplify the model, these simulations do not take into account the rapid change in membrane conductivity which occurs when cells become electroporated by pulsed electric fields. Incorporating these changes in conductivity would require us to experimentally quantify the dielectric response of the cell membrane and nuclear envelope to the HFIRE pulse parameters used, which goes beyond the scope this study.

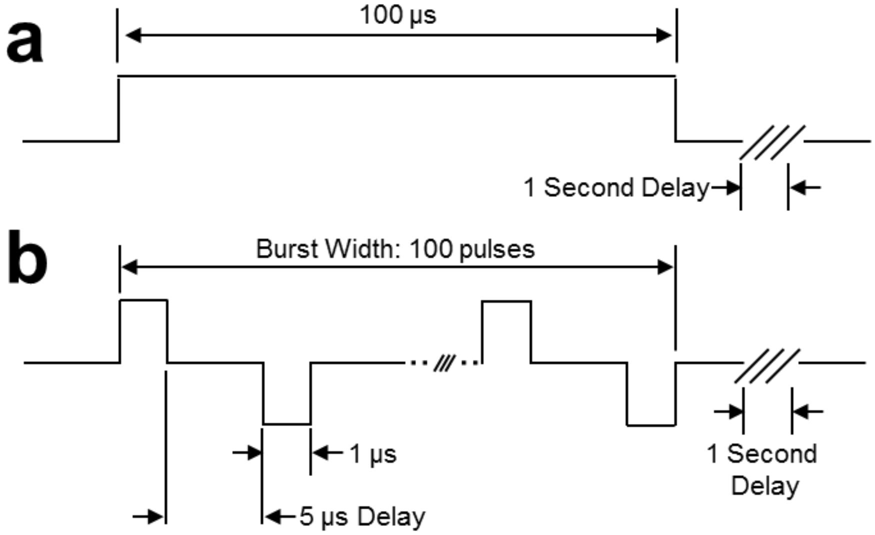

Supplementary Figure 3. Schematic of IRE pulse and HFIRE bursts (**a**) The IRE therapy delivers a series of mono-polar pulses which are 100 µs in duration. (**b**) In HFIRE, the mono-polar pulse is replaced by a rapid burst of 100x bi-polar pulses 1 µs in duration. A 5 µs delay in between alternating pulses is used to protect the electronics from ringing.

Supplementary Table 1: *Physical properties used in finite element models of hydrogel treatments. * measured values, ‡ default material values in COMSOL*

Parameter	Symbol	Value	Unit	Reference
IRE Voltage	V_{IRE}	450	[V]	*
H-FIRE Voltage	V_{HFIRE}	450-700	[V]	*
Electrode Density	ρ_e	7850	[kg/m^3]	‡
Electrode Specific Heat Capacity	Cp_e	475	[J/(kg·K)]	‡
Electrode Thermal Conductivity	k_e	44.5	[W/(m·K)]	‡
Electrode Conductivity	σ_e	4.03×10^6	[S/m]	‡
Electrode Permittivity	ε_e	1		‡
Hydrogel Density	ρ_h	997.8	[kg/m^3]	[18]
Hydrogel Specific Heat Capacity	Cp_h	4181.8	[J/(kg·K)]	[18]
Hydrogel Thermal Conductivity	k_h	0.6	[W/(m·K)]	[18]
Hydrogel Conductivity	σ_h	1.2	[S/m]	[18]
Hydrogel Permittivity	ε_h	0		[18]

Supplementary Table 2: *Physical properties used in finite element models of single cells. * measured values, ‡ approximation based on water composition*

Parameter	Symbol	Value	Units	Reference
Media Conductivity	σ_m	0.98	[S/m]	*
Media Permittivity	ε_m	$80\varepsilon_0$	[F/m]	‡
Cytoplasm Conductivity	σ_{cyt}	0.3	[S/m]	42
Cytoplasm Permittivity	ε_{cyt}	$154.4\varepsilon_0$	[F/m]	43
Nucleoplasm Conductivity	σ_{nuc}	1.35	[S/m]	42
Nucleoplasm Permittivity	ε_{nuc}	$52\varepsilon_0$	[F/m]	42
Cell Membrane Thickness	t_{mem}	5×10^{-9}	[m]	44
Nuclear Membrane Thickness	t_{Nmem}	40×10^{-9}	[m]	42
Cell Membrane Conductivity	σ_{mem}	3×10^{-7}	[S/m]	45
Cell Membrane Permittivity	ε_{mem}	$8.57\varepsilon_0$	[F/m]	46
Nuclear Membrane Conductivity	σ_{Nmem}	6×10^{-3}	[S/m]	42
Nuclear Membrane Permittivity	ε_{Nmem}	$28\varepsilon_0$	[F/m]	42
Domain Side Length	L_d	300×10^{-6}	[m]	-
Benign Cell Length	L_c	60×10^{-6}	[m]	*
Benign Cell Width	w_c	20×10^{-6}	[m]	*
Benign Nuclear Length	L_n	19.7×10^{-6}	[m]	*
Benign Nuclear Width	w_n	6.2×10^{-6}	[m]	*
Malignant Cell Length	L_c	120×10^{-6}	[m]	*
Malignant Cell Width	w_c	20×10^{-6}	[m]	*
Malignant Nuclear Length	L_n	20.4×10^{-6}	[m]	*
Malignant Nuclear Width	w_n	14.7×10^{-6}	[m]	*

Supplementary Note 1: Finite element models for electric field distributions.

The electric field distribution was found by solving the Laplace Equation:

$$\nabla^2 \phi = 0 \qquad (4)$$

where ϕ is the electrical potential. The boundaries of one electrode were set to the applied voltage (ϕ = $V_{applied}$) and the boundaries of the second were set to ground (ϕ = 0) while the initial voltage (V_0) for all subdomains were set to 0V. All other external boundaries were set to electrical insulation ($-\boldsymbol{n} \cdot \boldsymbol{J} = 0$).

Supplementary Note 2: HFIRE and IRE joule heating considerations.

Thermal effects on the scaffolds due to resistive losses were modeled by solving the Joule heating equation:

$$\nabla \cdot (k\nabla T) + \sigma|\nabla\phi|^2 \cdot \frac{d}{\tau} = \rho c_p \frac{\delta T}{\delta t} \qquad (5)$$

where k is the thermal conductivity, T is the temperature, c_p is the specific heat capacity, and ρ is the density of the 3D scaffold. $\sigma|\nabla\phi|^2$ is the Joule heating term, which was simplified by using a scaling factor proportional to the ratio of pulse duration d and pulse interval τ. This duty cycle approach, in which the total energy delivered to the tissue is averaged throughout the duration of the treatment, has been shown to accurately reproduce experimental results in hydrogels[17] and tissue[31]. Provided that the upper most boundary (perpendicular to electrodes) was exposed to the environment, it was assigned convective cooling properties $(-n \cdot (-k\nabla \mathrm{T}) = h(T_{ext} - T)$ with a coefficient value, h, of 50 $W/m^2{\cdot}K$ [31] while the remaining external boundaries of the simulation domain were set to thermal insulation $(-n \cdot (-k\nabla \mathrm{T}) = 0)$. The initial temperatures for all domains as well as the exterior temperature were set to 20°C.

References

42. Asami, K., Takahashi, Y. & Takashima, S. Dielectric-Properties of Mouse Lymphocytes and Erythrocytes. *Biochim Biophys Acta* **1010**, 49-55 (1989).
43. Yang, J. et al. Dielectric properties of human leukocyte subpopulations determined by electrorotation as a cell separation criterion. *Biophys J* **76**, 3307-3314 (1999).
44. Gascoyne, P.R.C., Pethig, R., Burt, J.P.H. & Becker, F.F. Membrane-Changes Accompanying the Induced-Differentiation of Friend Murine Erythroleukemia-Cells Studied by Dielectrophoresis. *Biochim Biophys Acta* **1149**, 119-126 (1993).
45. Sano, M.B., Henslee, E.A., Schmelz, E.M. & Davalos, R.V. Contactless dielectrophoretic spectroscopy: Examination of the dielectric properties of cells found in blood. *Electrophoresis* **32**, 3164-3171 (2011).
46. Alberts, B. et al. Molecular Biology of the Cell (3rd edn). *Trends in Biochemical Sciences* **20**, 210-210 (1995).
